# Constrained unsteady-state iris fast certification for lightweight training samples based on the scale change stable feature and multi-algorithm voting

**DOI:** 10.1371/journal.pone.0232319

**Published:** 2020-05-05

**Authors:** Shuai Liu, Yuanning Liu, Xiaodong Zhu, Jingwei Cui, Qixian Zhang, Tong Ding, Kuo Zhang, Zukang Wu, Yanan Yang

**Affiliations:** 1 College of Computer Science and Technology, Jilin University, Changchun, China; 2 Key Laboratory of Symbolic Computation and Knowledge Engineering of Ministry of Education, Jilin University, Changchun, China; 3 College of Software, Jilin University, Changchun, China; Chang Gung University, TAIWAN

## Abstract

Aiming at the problem of fast certification for a constrained iris in the same category caused by the unstable iris features caused by the change of the iris acquisition environment and shooting status under lightweight training samples, a one-to-one fast certification algorithm for constrained unsteady-state iris based on the scale change stable feature and multi-algorithm voting is proposed. Scale change stable features are found by constructing an isometric differential Gaussian space, and a local binary pattern algorithm with extended statistics (ES-LBP), the Haar wavelet with over threshold detection and the Gabor filter algorithm with immune particle swarm optimization (IPSO) are used to represent the stable features as binary feature codes. Iris certification is performed by the Hamming distance. According to the certification results of three algorithms, the final result is obtained by multi-algorithm voting. Experiments with the JLU and CASIA iris libraries under the iris prerequisite conditions show that the correct recognition rate of this algorithm can reach a high level of 98% or more, indicating that this algorithm can improve the operation speed, accuracy and robustness of certification.

## 1. Introduction

Aiming at a one-to-one category certification scenario with a lightweight training samples, a one-to-one fast certification algorithm for constrained unsteady-state iris based on the scale change stable feature and multi-algorithm voting is proposed.

The prerequisite conditions for the use of this method are as follows:

1. The tester collects the iris under the premise of the collection posture guidance, which allows the head of the iris to look directly at the camera, avoiding the interference of iris rotation;
2. Because the collected iris cannot control the distance between the person and the camera, it will be disturbed by defocus.
3. The infrared camera is used to collect the grayscale image of the iris, so there is a problem that the infrared light source is unevenly distributed in the image;
4. The number of training samples of one category is lightweight (only up to several hundred).

Taking multi-algorithm decision-making as the basic method, a one-to-one fast certification algorithm for constrained state long interval iris shooting based on a scale change stable feature and multi-algorithm voting is proposed in this paper. First, to improve the speed of iris certification, the scale change stable feature (SCSF) is designed by simplifying the SIFT, which is only used to find the template iris's stable feature points, and the Hamming distance is used as the recognition method, thereby improving the speed of iris certification under the same conditions. Stable feature points are taken as test objects, the local binary pattern algorithm with extended statistics (ES-LBP), the Haar wavelet with over threshold detection, and the Gabor filter algorithm with immune particle swarm optimization (IPSO) are used to represent the stable features as binary feature codes, which can improve the difference of iris codes of different categories on the basis of code length reduction. The final conclusion is obtained by voting on the certification results of three algorithms, which can accelerate speed while improving the certification accuracy and suppress the interference of texture state change due to unsteady iris caused by defocus and lighting effects.

**Fig 1:**
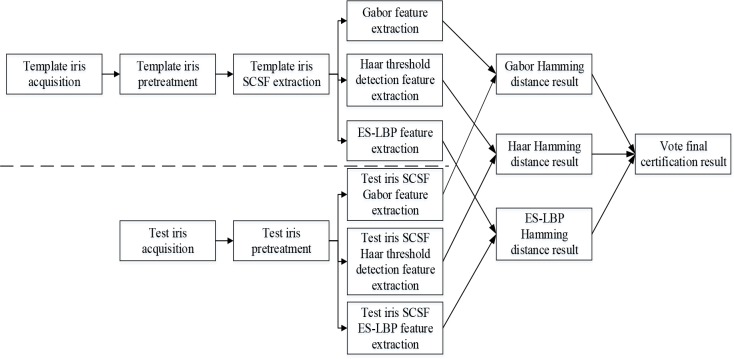
The overall work process of the algorithm.

The overall work process of the algorithm in this paper is shown in Fig 1.

The innovation and research significance of the algorithm in this paper are:

1. Taking into account the premise of the state change caused by constrained unsteady Iris, the instability is transformed into a stable state by extracting the features of the stable feature points, thereby avoiding the problem of different states feature expression chaotic, while reducing feature redundancy.
2. Through three different feature authentication algorithms to vote, avoiding possible disadvantages of a single algorithm in a specific environment, and to maximize the algorithm's tolerance to environmental conditions and the range of iris certification.
3. Execute certification by Hamming distance, and then reduces the training process and certification time of multi-algorithm feature recognition, avoids excessive training, and improves certification time under the same conditions.

## 2. Background and related work

Iris recognition is one of the most recognized technologies in the biometric recognition field, the foundation of which is feature extraction and recognition [1]. In the current application scenario, there is a case in which the same category comparison that tests features is compared with certification information previously stored in the chip card. This type of traditional approach is template matching mode. The human iris refers to the annular band between the sclera and the pupil; at present, the mainstream research direction of template matching mode is that through statistical learning, the distribution law of iris texture is extracted and then certified.

The texture distribution feature extraction algorithms are mainly based on statistical ideas, The feature extraction algorithms are mainly divided into three categories: the filter-based method, such as Daugman's multi-scale two-dimensional Gabor filter [2], and various improved filters based on it [3–5]; the image transformation method, such as wavelet transform and SIFT[6–8]; and the spatial domain to extract iris features, such as the local binary pattern method, which explores the relationship among the grey value of iris texture or expresses iris features through blocks and pits [9–11].

The current mainstream method in the one to one recognition algorithms are mainly divided into a distance class algorithm and a neural network class algorithm. The distance class algorithm is represented by the Hamming distance [3]. The distance class algorithms are suitable for iris recognition of a small number of categories with large differences in features because they omit the statistical learning process in recognition. The neural network [8] class algorithms are suitable for multi-category classification with a large number of categories because they can train data through statistical learning, which can improve recognition accuracy. However, the neural network requires the support of high-quality sample data, and the structural adjustment process is complicated.

At present, there are two main prerequisites for affecting the recognition effect of the iris recognition algorithm. One is the iris algorithm, and the second is the quality of the collected iris, which includes the external environment of iris collection and the state of the collection person. The external environment includes illumination and equipment parameters; the state of the collection person includes the degree of head deflection, defocus, blinking and acquisition constrained. At present, the prerequisites for iris training of most iris certification studies are mainly the ideal iris in the mode where the acquisition interval is short (adjacent frames), the external shooting environment is stable, and the acquisition state is very standard, which allows the various feature extraction methods to obtain good results in a variety of certification experiments but have some problems in practical applications.

1.**Uncertainty of unsteady iris on feature expression:** Most training and learning recognition models in the current iris recognition algorithms are based on publicly known iris training sets. However, during the actual shooting, for the same person, there is no guarantee that the acquisition status is the same at different time(especially at long time intervals), even if the acquisition process has external collection posture guidance constraints and auxiliary guidance, which may result in the test iris having some differences compared with the template iris, and leads to instability in the feature expression. Unlike humans and fingerprints, which have clear definitions of features, the iris itself has no relatively clear definition. The process of iris recognition is more dependent on the digital representation of image features and the advantages of feature recognition methods.Therefore, in order to improve the accuracy of recognition, in iris recognition of template matching mode, it is extremely important to test that the feature expressions of the iris and template iris are consistent, which needs to address the effects of unsteady-state iris.

**Fig 2:**
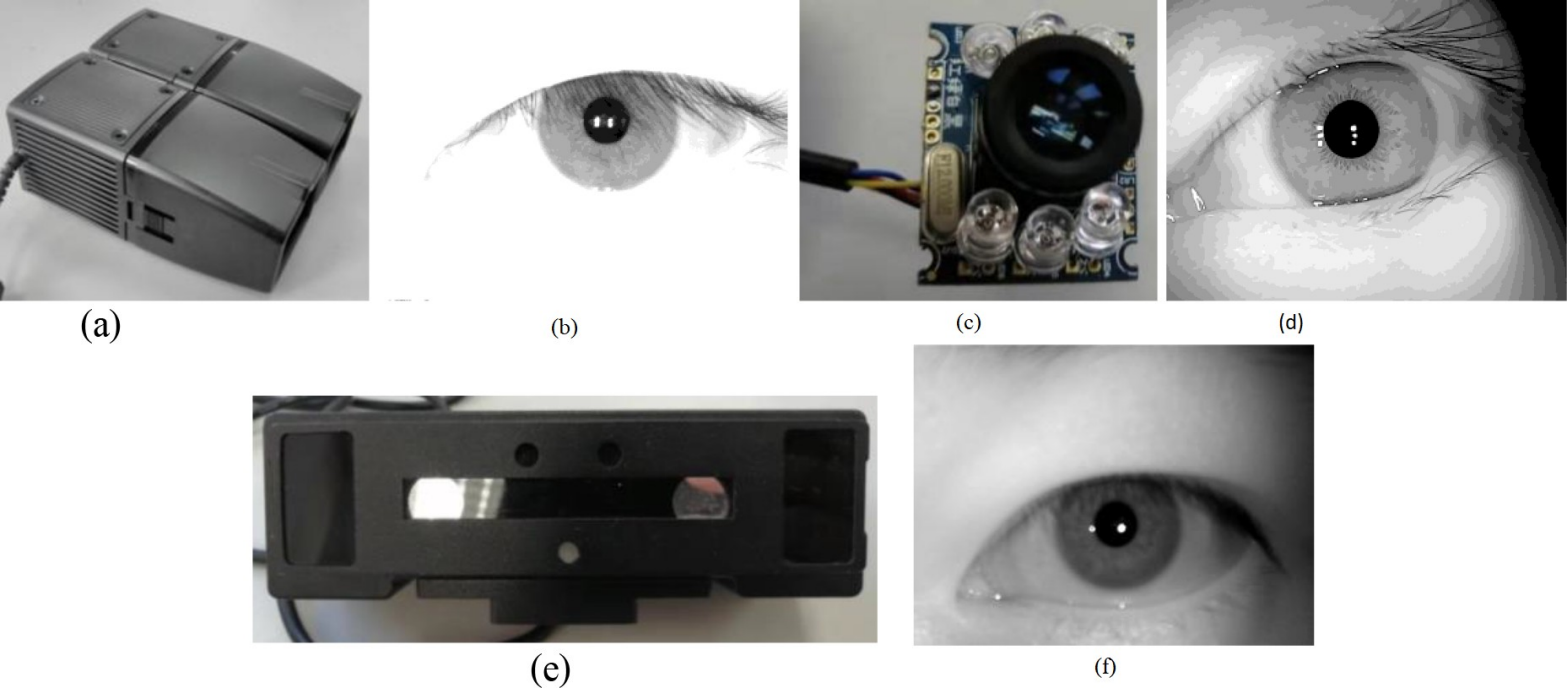
Eye acquisition sensor(a) NIR acquisition sensor, (b) NIR acquisition sensor for ideal images, (c) Ordinary optical acquisition sensor, (d) Ordinary optical acquisition sensor for ideal images, (e) Ordinary optical acquisition sensor for upgrading sensors, (f) Ordinary optical acquisition sensor with upgraded sensors corresponds to ideal images.

There are many reasons for the unsteady, and they are divided into three main aspects:

- The acquisition sensor specifications are different (e.g., an NIR device [12] and ordinary optical camera [13]; iris sample pictures taken by different cameras are shown in Fig 2).
- The collection environment is different because the iris status is unstable under different environments (illumination, etc.), which affects the relative relationship between the iris textures.
- The collection status of people is different: according to whether there is a collection posture guidance when collecting, it can be divided into the existence of a constrained state (with collection posture guidance) and an unconstrained state (without collection posture guidance).Because the collection status of people at different moments (especially with long time intervals) is unpredictable, in the unconstrained state, there are many kinds of collection conditions of the camera, which makes the status of the collected images diverse. Even in a constrained state, the specifications of the iris image have some standards, and there will be interference from issues such as defocus (the distance of the person from the camera), deflection (the deflection of the eye), and illumination (natural light, infrared light).

**Fig 3:**
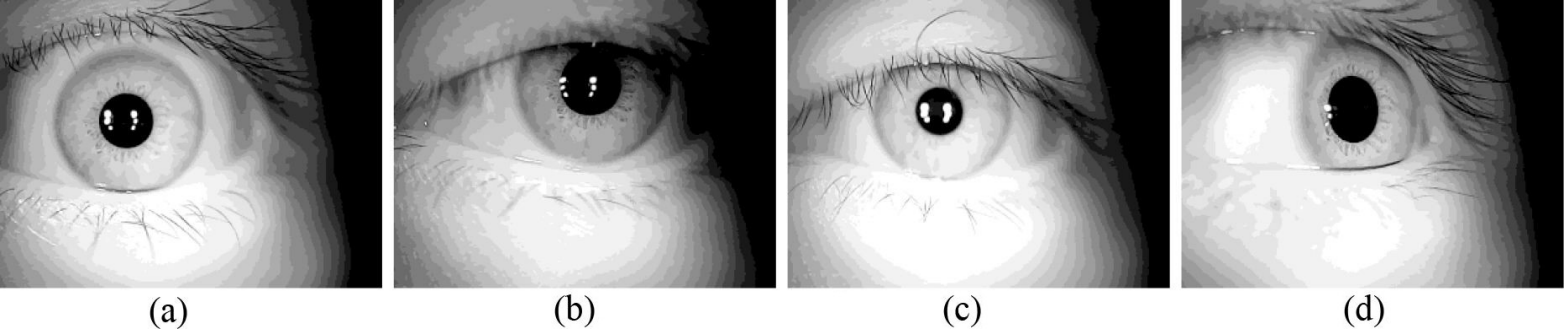
An example of a multi-state iris, (a)normal iris, (b)iris with dark condition, (c)iris with defocused condition, (d)iris with deflected condition.

Because the algorithm applied in the process of iris processing and recognition takes on a black box state, it is impossible to predict the outcome, which makes it difficult to design a unified process for a variable and unsteady iris using fixed parameters and to subsequently form selected effects for iris quality, localization and feature expression; this difficulty leads to the unsteady nature of iris features. An example of multi-state irises (taking the same device and the same person as an example) is shown in Fig 3.

2. **Defects of traditional recognition methods:** The current common iris feature extraction and recognition algorithms for one-to-one certification also have certain defects: the feature extraction algorithms of the spatial domain require high-quality images, and weak changes may also lead to the transformation of spatial features. Second, the operation steps of image transformation are too complicated. The SIFT method is taken as an example; although it has good scale-independent and anti-noise ability, it is not suitable for fast certification due to a large number of calculations. Although the filters methods are currently the most commonly used, there are many factors that need to be considered in parameter training, and the process of designing a more complete parametric training model is too complicated.

In response to the above-mentioned problems, some improved algorithms for iris feature extraction and recognition have been proposed:

For the uncertainty of unsteady iris on feature expression, there are currently some improved algorithms proposed: For example, the image processing algorithm is used to convert the multi-state iris to a stable state iris to extract iris features [14]; set the iris label for the statistical cognitive learning method of unclassified mixed data and recognize by convolutional neural network [15]; For the label modification of the existing neural network architecture, an error correction code-based label optimization method [16] is proposed to classify the iris state to better train the recognition model; Remind users to perform category label retraining with feedback mechanism through data accumulation feedback [17];

For the defects of traditional recognition methods, at present, there are DeepIris [18] and DeepIrisNet [19], which are specialized in iris recognition. DeepIrisNet [20], a iris recognition framework for transfer learning of lightweight training samples. And design the overall process of the iris through the method of multi-algorithm decision-making [21] to improve the recognition correct rate. Multi-feature fusion is used to identify features through weighting [6].

These methods have very good results, but they also have certain defects. Deep learning-based pattern recognition provides a powerful research direction for iris recognition. However, in the actual recognition process, the limitations of the existing iris public dataset size and situation classification are difficult to meet the needs of learning methods under a single deep learning framework Data volume requirements. This makes it difficult for deep learning models to work directly with light-weight training samples with insufficient classification and greatly limits the learning process of the iris recognition model. Not only that, many deep learning frameworks have certain requirements on computing equipment, which increase the cost of training. Therefore, in order to reduce the calculation cost, the traditional template matching pattern recognition method is more suitable. In addition, for the one-to-one certification scenario, the neural network method is too complicated and has too much parameters need to be adjusted, which is not suitable for rapid certification and training.

## 3. Materials and methods

### 3.1. Iris image processing

**Fig 4:**
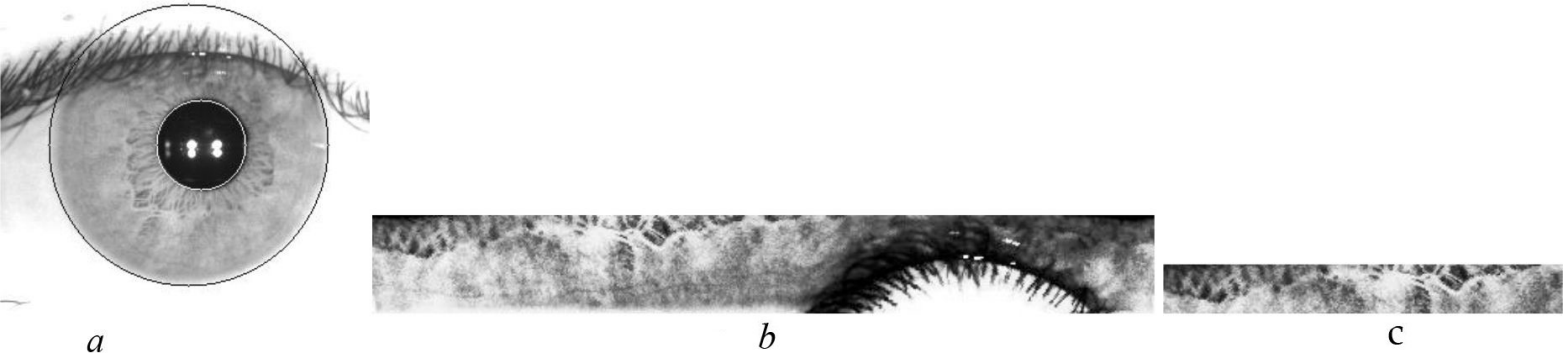
Iris processing: (a) iris segmentation image, (b) iris normalized and enhanced image, (c) iris recognition area.

The iris image needs to be processed before extracting the feature. Iris images(640×480 dimensional) in which the recognition process cannot be completed are eliminated by quality evaluation [22]. The iris region is segmented [23] and mapped to a 512×64 dimensional normalized image by Daugman rubber band normalization [24]. The texture in the normalized image is highlighted by equalizing the histogram [25], and a 256×32 dimensional recognition area is cut from the upper left corner. The process of iris processing is shown in Fig 4. In this paper, iris certification is carried out under the premise that iris inner and outer circles are correctly segmented. To ensure that iris quality and positioning will not affect iris certification.

**Fig 5:**
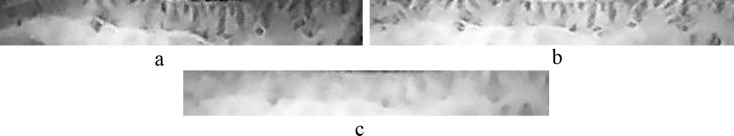
The same person's iris recognition areas in different shooting states: (a) clear greyscale image with darker light condition, (b) clear greyscale image with normal light condition, (c) blur greyscale image with normal light condition.

The example of the same person's iris recognition areas in different shooting states is shown in Fig 5.

It can be seen from Fig 5 that after treating the same person’s irises, although the overall contours are similar, differences also exist between the sharpness and specific features, which will affect the feature expression.

### 3.2. Scale change stable feature

Because the algorithm in this paper is designed for the constrained iris with a long shooting interval, the most difficult problem affecting the certification accuracy is limited to the defocus and external illumination problems during shooting. Phase problems such as iris rotation can be ignored. Therefore, to make the selection of iris feature points faster and speed up certification, the scale change stable feature (SCSF) is designed by simplifying the traditional SIFT.

The scale change stable feature (SCSF) refers to the feature point that can still obtain a good detection effect after a series of image reduction and enlargement processes. Images in each layer are adjusted to the same dimensions by constructing an equal dimension difference Gaussian space. The points that can still be detected after the Gaussian filtering process are found and taken as stable feature points.

The specific steps are:

1. The original image dimension is 256×32. First, adjust the dimensions of the image by 2 × 2 average pooling [26] to build a 5-layer pyramid. The dimensions of the first layer are 512×64, the dimensions of the second layer are 256×32, and the dimensions of the third layer, fourth layer and fifth layer are 128×16, 64×8, and 32×4, respectively. Each layer is processed with five different Gaussian filters [27].The Gaussian filtering *G*(*x*,*y*) formula is shown in Formula (1).
  $$G(x,y)=\frac{1}{2\pi {\left(k\times \sigma \right)}^{2}}e^{-\frac{x^{2}+y^{2}}{2{\left(k\times \sigma \right)}^{2}}}\;k=1,2,3,4,5$$ (1)
  Each layer has five images, the smoothing factor *σ* of the first image in each layer is set to 5, and then the smoothing factor of each image is multiplied by *k* to form a new smoothing factor *k*×*σ*.
2. Two adjacent images in each layer are subtracted one by one to form a differential image layer, and each layer has four differential images. After the differential image is obtained, the interpolation method [28] is used to unify adjust the dimensions in the differential images of each layer to 256×32.
3. Filter processing values in the four images subjected to the same Gaussian filter in the five layers are read, and then four groups of filter processing values are compared. The point whose values are all not 0 in the four groups is a stable feature point.
4. The scale change stable features consist of all stable feature points, and the total is *T*.

The purpose of extracting stable feature points in this way is to reduce the number of feature points that need to be detected. In this paper, the factors that affect the appearance of iris features are limited to two conditions: defocus and illumination distribution, which makes the phase difference between the test iris and template iris negligible and feature points can be mapped one-to-one between the same locations,which premise lays the foundation for the calculation method of feature points in this paper.

Through different Gaussian filter and scale changes to stabilize the characteristics, the points of the iris texture edges are mainly selected. There will be a large difference in pixel values between these points and the surrounding pixels, which will be less affected by the defocus and uneven light distribution. In this state, there can still be an irreversible difference between the values of the pixels in the surrounding neighborhood. The value of the difference may change, but the difference between the texture edge points and the surrounding neighborhood points will not change (If the gray value of the texture edge feature points in the template iris is greater than the surrounding pixel values, the gray value of the texture edge feature points of the test iris will also be greater than the surrounding pixel values), which makes the feature points very stable and powerful.

The grayscale relationship between the selected feature points and the surrounding pixels is stable, and it is not easy to change due to changes in the state and environment, and such feature points are also likely to resonate with the feature extraction algorithm, which is also conducive to the transformation of the binary feature code. In addition, this point all exists in the same phase of the test iris and template iris, and represents the same iris position, which is helpful for the feature extraction algorithm to convert it from image points to digital features, reducing unnecessary redundancy.

### 3.3. Iris feature extraction and recognition

#### 3.3.1. ES-LBP

**Fig 6:**
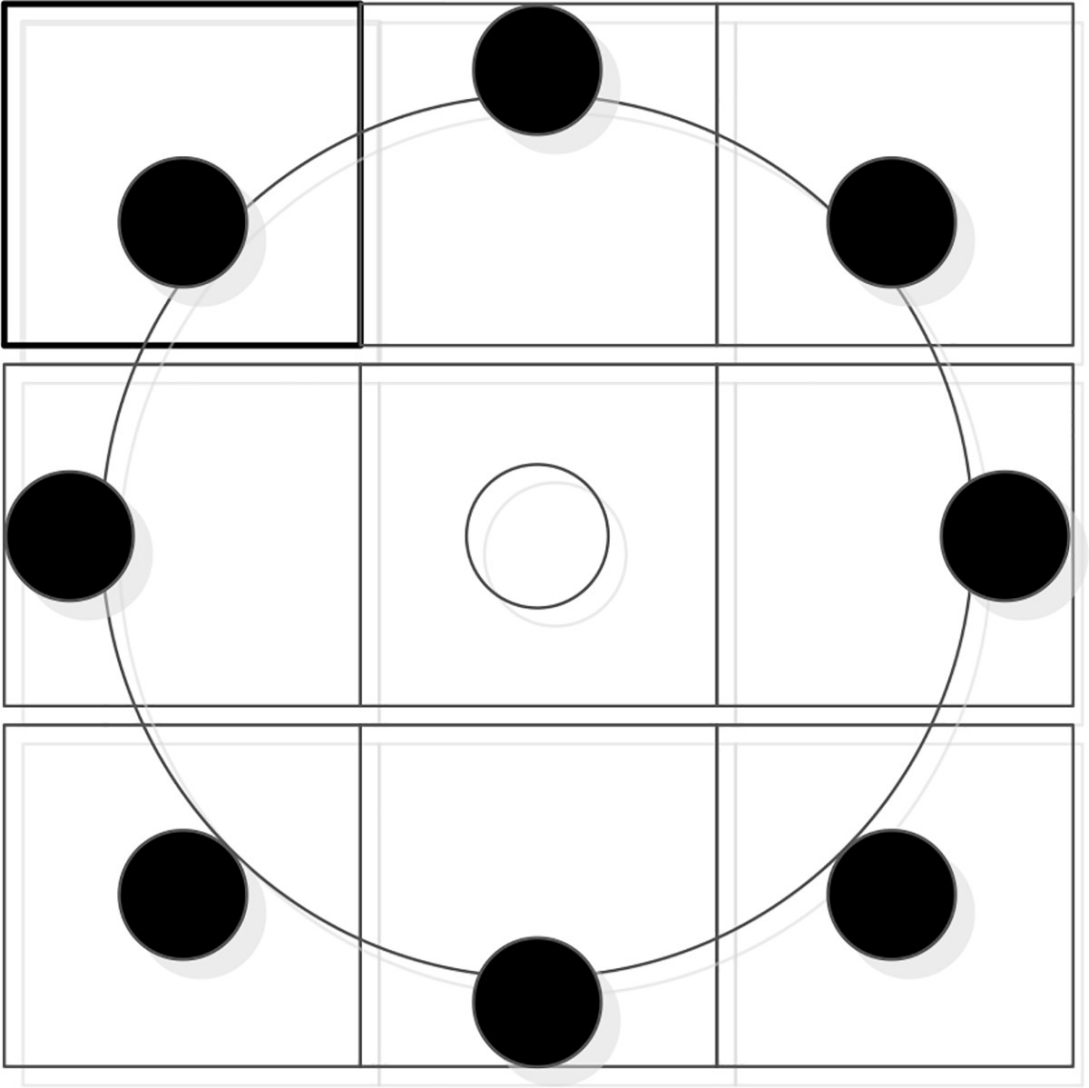
Circular neighbourhood schematic diagram.

The traditional LBP operator is a 3×3 neighbourhood space, although the experimental effect is also well-defined; a small range cannot accurately represent texture at different scales and frequencies. To adapt to texture features with different scales and frequencies, the circular neighbourhood is used to replace the square neighbourhood, which can extend the LBP operator neighbourhood. The improved LBP operator allows any number of pixels in a circular neighbourhood of radius R [29]. A circular neighbourhood with a radius of 3 with eight pixel points is taken as the example. The schematic diagram is shown in Fig 6.

The extended statistical LBP algorithm (ES-LBP) on the basis of a circular neighbourhood is proposed in this paper. With each stable feature point as a centre, *N* circular neighbourhoods are established, and each of neighbourhoods has a different number of pixel points. In each circular neighbourhood, the grey value of the pixel point is compared with the grey value of the centre point. If the grey value of the pixel point is larger than the grey value of the centre point, the pixel point is represented by 1; otherwise, the pixel point is represented by 0. Each feature point is represented by an *N*-bit binary feature code, and the total is *N*×*T* bits. The feature code of each feature point is set according to the number of 1s in the circular neighbourhood. The ordering rule of the feature codes is that the radius of the circular neighbourhood is from small to large if the radius of the circular neighbourhood is the same in ascending order of pixel points from fewer to more. If the number of 1s in the circular neighbourhoods is greater than one half of the number of feature points in the neighbourhood, the feature code is 1; otherwise, it is 0. The binary feature code representation with ES-LBP is shown in Formula (2).

$$ES-LBP_{N,R}=\left\{ \begin{array}{l} 1\;\sum_{i=0}^{R}Sgn(I_{i}-I_{O})\geq (N/2)\\ 0\;\sum_{i=0}^{R}Sgn(I_{i}-I_{O})<(N/2) \end{array}\right.$$ (2)

Among: *I*_*i*_ is the pixel value of the i-th comparison pixel point of the iris, and *I*_*o*_ is the pixel value of the center point corresponding to the comparison pixel point.

*ES−LBP*_*N*,*R*_ represents the *N-th* feature code, which is the representative value of *R* pixel points in the *N-th* circular neighbourhood.

Considering the reason for dimension reduction and time complexity, ten circular neighbourhoods are established at each feature point. The radius of each group is selected from 1, 2 and 3. The corresponding relationship among the radius and the number of feature points is (1,4), (1,8), (2,8), (2,12), (2,16), (2,20), (3,12), (3,16), (3,20), (3,24). The final texture feature is transformed into a 10×*T* binary feature code.

#### 3.3.2. Haar wavelet with over threshold detection

**Fig 7:**
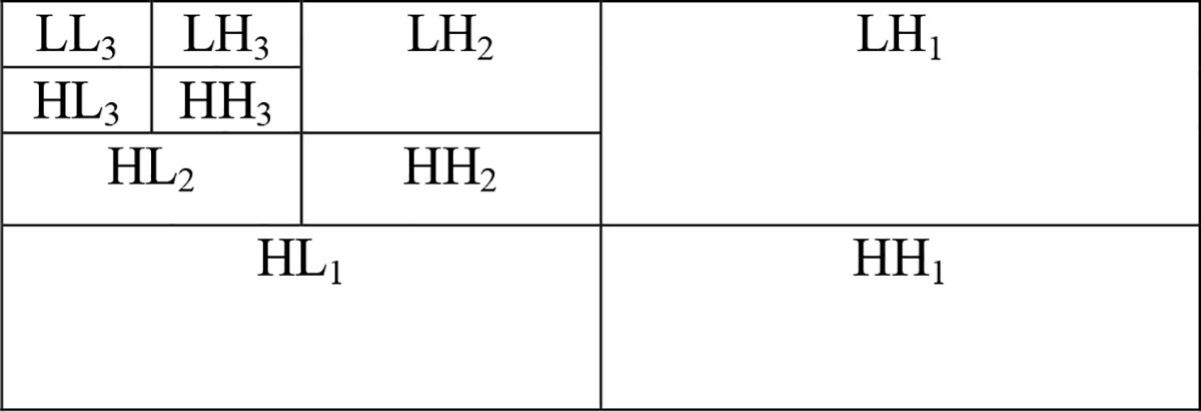
Haar wavelet decomposition diagram.

Haar wavelet processes an iris normalized image from high to low scale. *LL*_*i*_,*HL*_*i*_,*LH*_*i*_,*HH*_*i*_ are low-frequency sub-graphs, horizontal high-frequency sub-graphs, vertical high-frequency sub-graphs and diagonal high-frequency sub-graphs corresponding to the wavelet coefficient of layer *i* after image decomposition. The iris texture feature is rich, which is mainly reflected by high-frequency coefficients. However, if high-frequency coefficients in the first layer and the second layer are numerous, the code will be too long, and the space requirements will be too large, which affects the recognition accuracy. The number of high-frequency coefficients in the third layer is suitable for feature extraction [30]. Therefore, iris texture features are extracted from *LH*_3_,*HL*_3_,*HH*_3_. The Haar wavelet decomposition diagram is shown in Fig 7.

The third sub-block dimension is unified adjusted to 256×32. The high-frequency coefficients of three directions in each feature point are extracted in order of *LH*_3_,*HL*_3_,*HH*_3_, whose capacity is 3×*T*. According to the degree of approximation represented by the wavelet coefficients between decomposed the wavelet and the decomposed signal, it can be seen that the approximation of the positive wavelet coefficients and negative wavelet coefficients are different [31]. The over threshold detection method is used to encode high-frequency coefficients. The iris texture feature is set *S* = {*LH*_3_,*HL*_3_,*HH*_3_}. The code rule for feature element *S*(*i*) is as shown in Formula (3).

$$\{\begin{array}{c} S(i)=0\;S(i)\leq z\\ S(i)=1\;S(i)>z \end{array}$$ (3)

The feature element *S*(*i*) represents the value of the high-frequency coefficient at each point in the sub-block. *z* is the decision threshold, the value of z is the average of the values of the high-frequency coefficients of all feature points. If *S*(*i*)>*z*, 1 is used to represent this point. In contrast, if *S*(*i*)≤*z*, 0 is used to represent this point. In this way, the feature code is transformed into 3×*T* bit binary feature encoding.

#### 3.3.3. IPSO & Gabor filter

The expression of the Gabor filter is shown in Formula (4). [3]

$$\varphi (x,y)=\frac{f_{0}^{2}}{\pi \gamma \eta }e^{\left(-(\frac{f_{0}^{2}}{\gamma^{2}}x_{t}^{2}+\frac{f_{0}^{2}}{\eta^{2}}y_{r}^{2})\right)}e^{\left(-j2\pi f_{0}x_{r}\right)}$$ (4)

$$x_{r}=x\cos\theta +y\sin\theta ,\;y_{r}=-x\sin\theta +y\cos\theta$$

If, $y=\eta =\frac{\delta }{\sqrt{2\pi }}$, $\vec{k}=2\pi f_{0}e^{\left(j\theta \right)}$, $\vec{z}=(x,y)$. Formula (4) becomes as shown in Formula(5).

$$\varphi (\vec{z})=\frac{1}{2\pi }\frac{\|\vec{k}{\|}^{2}}{\sigma^{2}}e^{\left(-\frac{\|\vec{k}{\|}^{2}\|\vec{z}{\|}^{2}}{2\sigma^{2}}\right)}e^{{}^{\left(j\vec{k}\times \vec{z}\right)}}$$ (5)

To obtain a function in different directions *m* and frequency scale *n*, Formula(5) can be rewritten as:

$$\varphi_{m,n}(\vec{z})=\frac{1}{2\pi }\frac{\left\|\vec{k_{m,n}}\right\|}{\sigma^{2}}e^{{}^{\left(-\frac{\|\vec{k_{m,n}}{\|}^{2}\|\vec{z}{\|}^{2}}{2\sigma^{2}}\right)}}e^{\left(j\vec{k_{m,n}}\times \vec{z}\right)}$$

$$\vec{k_{m,n}}=k_{n}e^{{}^{\left(i\phi m\right)}},k_{n}=k_{\max}/f^{v},\phi_{m}=\pi m/8$$ (6)

In Formula (6), *ϕ*_*m*_ and *k*_*n*_ represent the direction and frequency of the Gabor filter, respectively, and standard deviation *σ* = 2*π*. *k*_max_ is the maximum frequency, *f*^*v*^ is the frequency difference between two adjacent Gabor nuclei, *v* = 1,2,3… *m*. Direction *φ* from 0° to 180° is divided into *n* parts. In this paper, *m* is set to 5, and *n* is set to 8; that is, the number of frequencies is five, the number of directions is eight, and the total number of Gabor filters is 40. The expression is determined by {*k*_max_, *f*^*v*^,m,n}.

The amplitude values of feature points are extracted by a Gabor filter with five frequencies and eight directions, and the filter with the largest amplitude of each feature point is found. The frequency and direction of the filter are numbered as 3-bit binary codes and spliced together in the order of frequency and direction. The features are turned into 6-bit feature codes, marked as *F*_*index−i*_. The total is a 6×T bit binary feature code. The encoding formula is shown in Formula (7).

$$F_{index-i}=\max(\varphi_{m,n}(\vec{z}))\;n=1,2,\ldots ,8\;m=1,2,\ldots ,5\;i=1,2,\ldots ,T$$ (7)

*F*_*index−i*_ denotes the feature code of the i-th feature point, $\varphi_{m,n}(\vec{z})$ denotes the feature point response amplitude in the m-frequency and n-direction.

The parameters of the Gabor filter are weighed against each other, and the parameters determine the quality of the filter effect. Therefore, the parameters of the filter need to be optimized.

In this paper, *k*_max_ and *f*^*v*^ are optimized using the immune particle swarm algorithm (IPSO), which integrates part of the immune algorithm [32] into the PSO algorithm [33], and 30 particles are used in PSO. Each particle has an initial velocity range of [–50,50]. Each particle contains a set of Gabor filter parameters to be optimized, which are equal to 30 initial Gabor filters. The initial value range of *k*_max_ is [0,2], *f*^*v*^ is [0,3]. The initial optimal individual initial *pBest* of a particle is set to the initial value, and the global optimal *gBest* is set to 0.

When parameters are optimized, one test iris, *j* training irises of the same category, and *j* training irises of different categories are taken. According to the distribution of feature points of the same category, the iris feature is extracted using a Gabor filter and converted into a binary feature code. The Hamming distance is used to calculate the similarity between the test iris and the training iris. After considering the concept of affinity in immune algorithms, the test iris can be seen as an antigen, and the training iris can be seen as an antibody. The affinity calculation formula is shown in Formula (8).

$$Q_{i}=1/(1+H_{i})$$ (8)

*Q*_*i*_ indicates the affinity of the test iris to the *i-th* training iris. *H*_*i*_ represents the Hamming distance between the test iris and the *i-th* training iris.

According to the principle of affinity, affinity in the same category of irises is high, and affinity among different categories of irises is low. The fitness formula of PSO is designed on the basis of this principle, which is shown in Formula (9).

$$QC=\sum_{s=1}^{j}Q_{s}/\sum_{t=1}^{j}Q_{t}$$ (9)

*Q*_*s*_ represents the affinity of the test iris and the *s-th* training iris in the same category, *Q*_*t*_ represents the affinity of the test iris and the *t-th* training iris in different categories. *QC* is the ratio of the sum of affinity in the same category, and the sum of affinity in different categories is calculated. The higher the affinity in the same category, and the lower the affinity in different categories, the larger *QC*. If the new *QC* is greater than the original *QC*, a new fitness *QC* is calculated. Then, new *pBest* are set as filter parameters corresponding to the new *QC*, and filter parameters corresponding to the maximum value of *QC* are set as new *gBest*. After new *pBest* and *gBest* are finalized, particles evolve according to Formula (10) and Formula (11).

$$v_{i}^{d}=\omega \times v_{i}^{d}+c_{1}\times rand_{1}^{d}\times (pBest_{i}^{d}-x_{i}^{d})+c_{2}\times rand_{2}^{d}\times (gBest_{i}-x_{i}^{d})$$ (10)

$$x_{i}^{d}=x_{i}^{d}+v_{i}^{d}$$ (11)

*ω* is the inertia weight, which is set to 0.729. *c*_1_ and *c*_2_ are acceleration factors, which are set to 1.49445 [33]. $rand_{1}^{d}$and $rand_{2}^{d}$ are random numbers in the [0,1] intervals.

**Fig 8:**
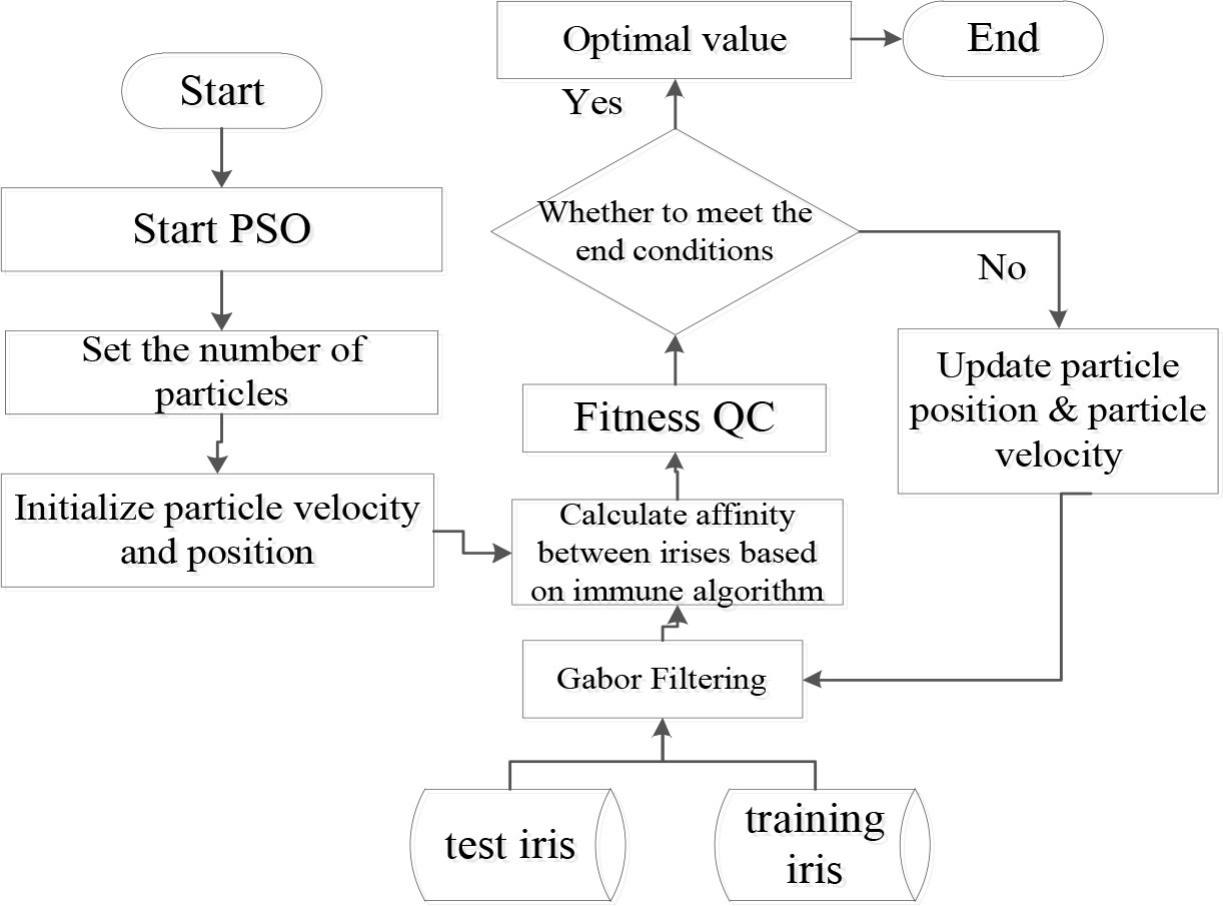
Gabor filter parameter optimization process.

If evolution does not reach the end condition, the evolutionary particle evolves again through 300 iterations, and parameters in the final *gBest* are used as parameters for the Gabor filter. The Gabor filter parameter optimization process is shown in Fig 8.

#### 3.3.4. Multi-algorithm voting

After obtaining the binary feature code of the three types of algorithms separately, the Hamming distances of three sets of feature codes between the test iris and contrast iris are calculated. The Hamming distance (HD) is calculated, as shown in Formula (12).

$$HD=\frac{1}{N}\sum_{i=1}^{N}A_{i}⊕B_{i}$$ (12)

Among: *A*_*i*_ indicates the feature code of the test iris, *B*_*i*_ indicates the feature code of the contrast iris. *N* represents the number of bits.

The classification thresholds *Y*_1_,*Y*_2_,*Y*_3_ of the three algorithms are obtained by statistically the results of the same category match and different category match of training iris. *Y*_1_ represents the classification threshold of ES-LBP; *Y*_2_ represents the classification threshold of Haar wavelet with over threshold detection; *Y*_3_ represents the classification threshold of Gabor filter method.

The role of the classification threshold is to meet the principle of the vast majority of iris training, which can effectively distinguish the same category from different categories of iris. Compare the calculated Hamming distance of the three algorithms with the corresponding classification threshold; if *HD* is less than the classification threshold of this algorithm it is assumed that test iris and contrast iris may belong to the same category. However, if *HD* is greater than or equal to the classification threshold of this algorithm, it is assumed that test iris and contrast iris belong to different categories.

If two or more of the three algorithms identify two irises as the same category, the two irises are ultimately identified as the same category. If two or more of the three algorithms identify two irises as different categories, the two irises are ultimately identified as different categories.

## 4. Experiment and analysis

**Experimental configuration:**The CPU used in all experiments is a dual-core 2.5 GHz, with 8 GB of memory and the Windows operating system.

**Experimental data:**The experimental iris library is the JLU iris library provided by the Biometrics and Information Security Laboratory of Jilin University [34] and the CASIA iris library provided by the Institute of Automation of Chinese Academy of Sciences [35]. The CASIA Iris Library provided by the Institute of Automation of the Chinese Academy of Sciences is one of the main iris libraries currently used in academia in the world. There are currently four versions (CASIA-V1, CASIA-V2, CASIA-V3, CASIA-V4), which contain multiple sub-libraries, and open libraries with tens of thousands of iris images. The JLU Iris Library provided by Jilin University's Biometrics and Information Security Technology Research Office is one of the iris libraries publicly available in Chinese universities. There are currently seven versions (JLU-1.0, JLU-2.0, JLU-3.0, JLU-4.0, JLU-5.0, JLU-6.0, JLU-7.0), hundreds of thousands of iris images in the complete library with hundreds of objects. The number is still increasing by 2020.

**Evaluation index:** ROC curve is the most commonly used iris recognition algorithm evaluation index.ROC curve represent the relationship between the false reject rate (FRR) and the false accept rate (FAR).The closer ROC curve is to axis, the better the algorithm performance.the value of when FRR and FAR are equal, is called the error rate (EER). The smaller EER, the better performance of iris recognition algorithm. In addition,correct recognition rate (CRR) is also one of indicators to evaluate the performance of iris recognition algorithms. [36] The correspondence between CRR, FAR, and FRR is shown in Formula group 13:

$$\mathrm{CRR}=\frac{\mathrm{Number}\;\mathrm{of}\;\mathrm{correct}\;\mathrm{matches}}{\mathrm{Total}\;\mathrm{number}\;\mathrm{of}\;\mathrm{matches}}$$

$$\mathrm{FAR}=\frac{\mathrm{Number}\;\mathrm{of}\;\mathrm{errors}\;\mathrm{accepted}}{\mathrm{Total}\;\mathrm{number}\;\mathrm{of}\;\mathrm{matches}\;\mathrm{between}\;\mathrm{classes}}$$

$$\mathrm{FRR}=\frac{\mathrm{Number}\;\mathrm{of}\;\mathrm{false}\;\mathrm{rejections}}{\mathrm{Total}\;\mathrm{number}\;\mathrm{of}\;\mathrm{matches}\;\mathrm{within}\;a\;\mathrm{class}}$$ (13)

**Experiment description:** The experiment in this paper is divided into four parts: "The meaning of algorithm", "Multi-algorithm Voting Specific Operation", "traditional algorithm comparison experiment", "time operation experiment". In all the experiments in this paper, training irises are completely different from testing irises.

Section 4.1 explains the meaning of the algorithm. The experiment is mainly to show the significance and rationality of the design of the experiment. It is divided into two parts. The Section 4.1.1 “Long Time Interval Shooting and Certification” shows the impact of iris texture non-steady state on recognition caused by long time interval shooting; The Section 4.1.2 “Significance of Multi-algorithm Voting and SCSF” can indicate the SCSF and multi-algorithm voting form set in this paper the rationality of the certification model. Section 4.2 is the specific operation of multi-algorithm voting. The operation process of the voting mechanism in multi-algorithm is explained through specific examples. Section 4.3 is a comparison experiment of traditional algorithms. There are some traditional algorithms for comparison under the same conditions, which illustrates the advantages of this paper. Section 4.4 is a time operation experiment, showing how the method in this paper reflects the characteristics of rapid certification.

### 4.1 The meaning of algorithm

#### 4.1.1.Impact of unsteady iris on certification

This experiment uses the iris composition in the JLU-6.0 iris library that meets the conditions set here to test the iris library. The test iris library contains 10 categories (each of which has 10 images for a total of 100). Use the iris image through the statistical form of the 8-neighborhood LBP operator [37], and count the number of pixels in the 256×32-dimensional iris image with the value of the center pixel greater than the value of the pixels in the neighborhood. If it is greater than or equal to four, it is set to 1, if it is less than four, it is set to 0, and the iris image is converted into a 254×30-bit binary feature code. The irises of the same category are matched with each other, and the impact of iris unsteady state on iris certification is analyzed by Hamming distance. A total of 100 matching comparisons were performed between iris in the same category and 200 matching comparisons between different categories of iris, with a total of 300 Hamming distance values. The impact of iris state uncertainty on long term interval shooting on iris certification is explained by the Hamming distance distribution of the same category and different categories.

List some Hamming distance data (including 5 Hamming distances for matching in the same category, and 15 Hamming distances for matching in different categories), is shown in Table 1. Among them, the serial number 1–5 is the Hamming distance value under the same category match, and the serial number 6–20 is the Hamming distance value under different categories match.

**Table 1:** Some Hamming distance data.

Serial number	Hammin distance	Serial number	Hammin distance	Serial number	Hammin distance	Serial number	Hammin distance
1	0.472743	6	0.463542	11	0.45434	16	0.474132
2	0.480035	7	0.480729	12	0.495313	17	0.472222
3	0.525	8	0.498264	13	0.499826	18	0.488889
4	0.481771	9	0.489757	14	0.45	19	0.467187
5	0.470139	10	0.490972	15	0.480035	20	0.496354

**Fig 9:**
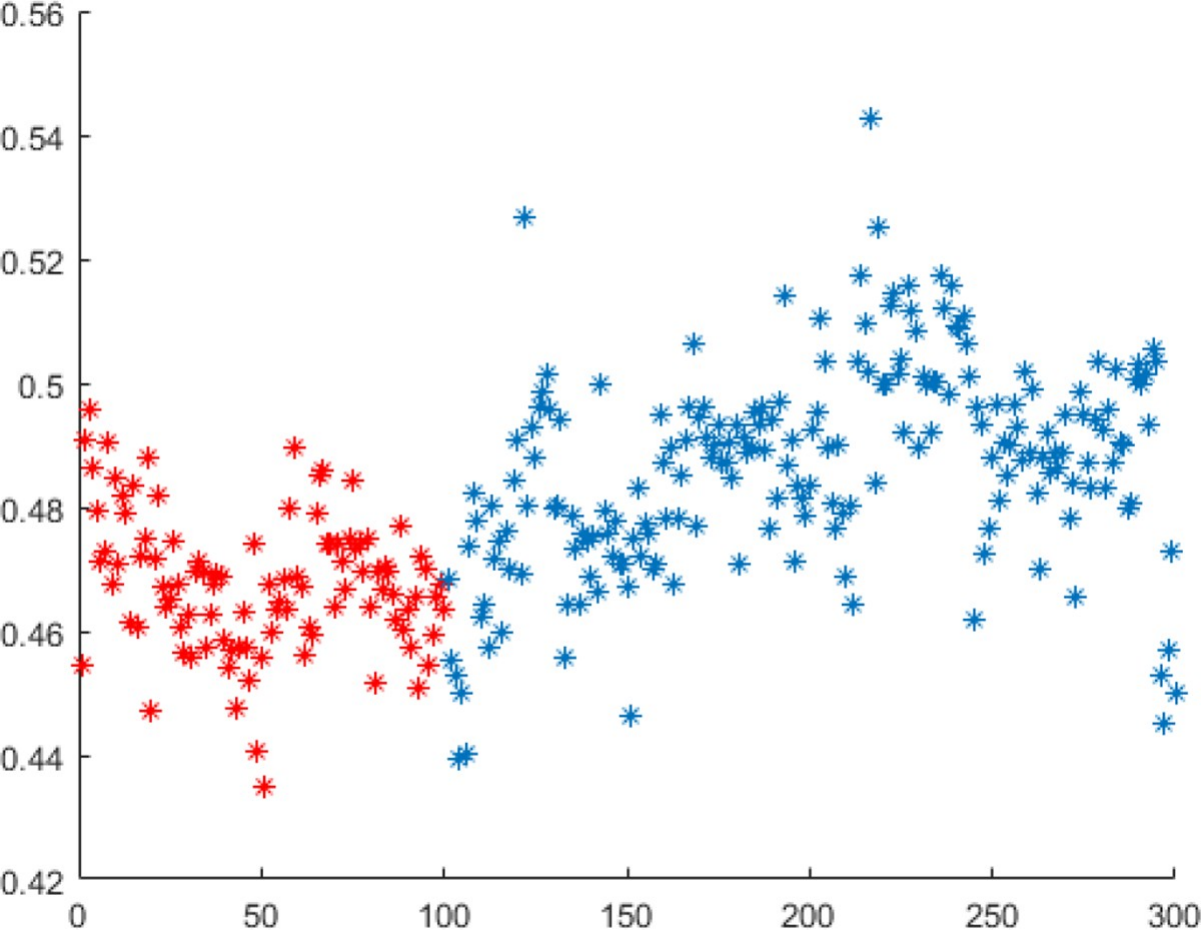
The distribution of 300 Hamming distance values.

The distribution of the 300 Hamming distance values is shown in Fig 9. (100 red points represent the Hamming distance values of the same category of iris matching comparison, and 200 blue points represent the Hamming distance values of the different categories iris matching comparison, abscissa (X axis) represents the image serial number, and the ordinate (y axis) represents the Hamming distance value)

In the template matching mode, the Hamming distance value of the same iris matching category should be less than 0.33. It can be seen from Table 1 and Fig 9 that the value of iris clustering is not in this range, and is usually between 0.4–0.6. If the unstable iris features are not converted into stable iris features by certain means, and the Hamming distance is directly used for feature expression and certification, although the Hamming distance values of different categories are the same as those of the same category, there will be some differences in the value of Hamming distance, but there are still situations where the difference in Hamming distance is not high and it is difficult to completely distinguish.

The reason for this is mainly because the collection status of the collecting person has changed, and it is impossible for humans to maintain the same status at different times to collect the iris. In this paper, the image is affected by light and defocus, and the appearance of its own texture features has changed. Hamming distance is a recognition method suitable for large differences. In the case of two images with defocus or lighting changes, the unclear texture and uneven lighting distribution will affect the gap between the texture pixels and surrounding pixels.The expression of the binary feature code will be affected (the difference relationship between pixels may change).

This situation has a more serious impact on non-textured areas, and excessively high binary signatures will also cause a large number of redundant and uncertain areas to be counted, affecting the certification effect, which makes the Hamming distance calculated in the experiments in this section is high.

Therefore, this article needs to select feature points, and choose texture edge points that have stable relationships with surrounding pixels and are not easily affected by defocus and light distribution as iris feature points.

#### 4.1.2. Significance of multi-algorithm voting and SCSF

It can be seen from 3.1.1 that the unsteady state iris caused by long time interval shooting of iris has an impact on the value of the Hamming distance. In order to eliminate such effects, this paper uses a multi-algorithm voting and SCSF stabilization feature method. This experiment explains The significance and rationality of these two settings. The irises are selected from the CASIA-V1 iris library that meet the conditions set in this paper to constitute the test iris library (120 categories and 5 images per category; a total of 600 images), which are used to test the performance of each algorithm. When testing, use the same number of test iris in the same category for the experiment (the test iris is different from the training iris). The test iris of the same category recognized each other 600 times, and the test iris of different categories recognized each other 2,400 times, a total of 3,000 times. The irises are selected from the CASIA-V4-Lamp, CASIA-V4-Interval iris library that meet this paper’s prerequisites to constitute the test iris library. The algorithm in this paper is compared with traditional iris recognition algorithms. The feature extraction range of the contrast experiment is the overall iris normalized enhancement image. The significance of multi-algorithm voting and SCSF can be analysed through these two experiments.

**Fig 10:**
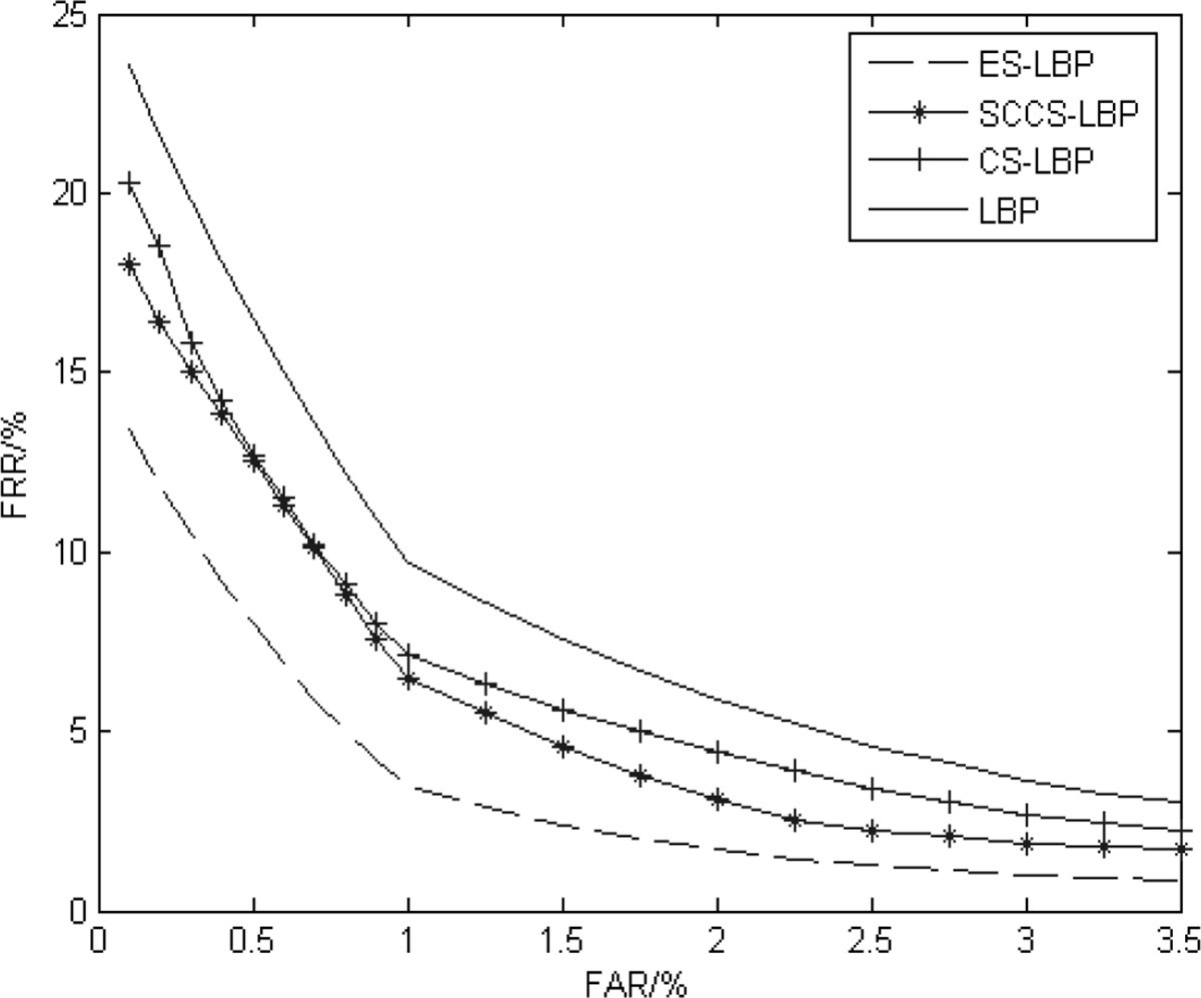
ROC curve of the LBP experiment.

**LBP algorithm:** The ES-LBP is compared with the statistical characteristic centre symmetric local binary pattern (SCCS-LBP) proposed in the literature [9], centre symmetric local binary pattern (CS-LBP)[38] and general local binary pattern (LBP). The Hamming distance is used for identification. The CRR and EER of different methods are shown in Table 2, and the ROC curve is shown in Fig 10.

**Table 2:** The CRR and EER of different methods in the LBP experiments.

Method	CRR	EER
ES-LBP	98.71%	1.84%
SCCS-LBP	97.74%	2.38%
CS-LBP	97.37%	2.84%
LBP	95.71%	3.25%

**Fig 11:**
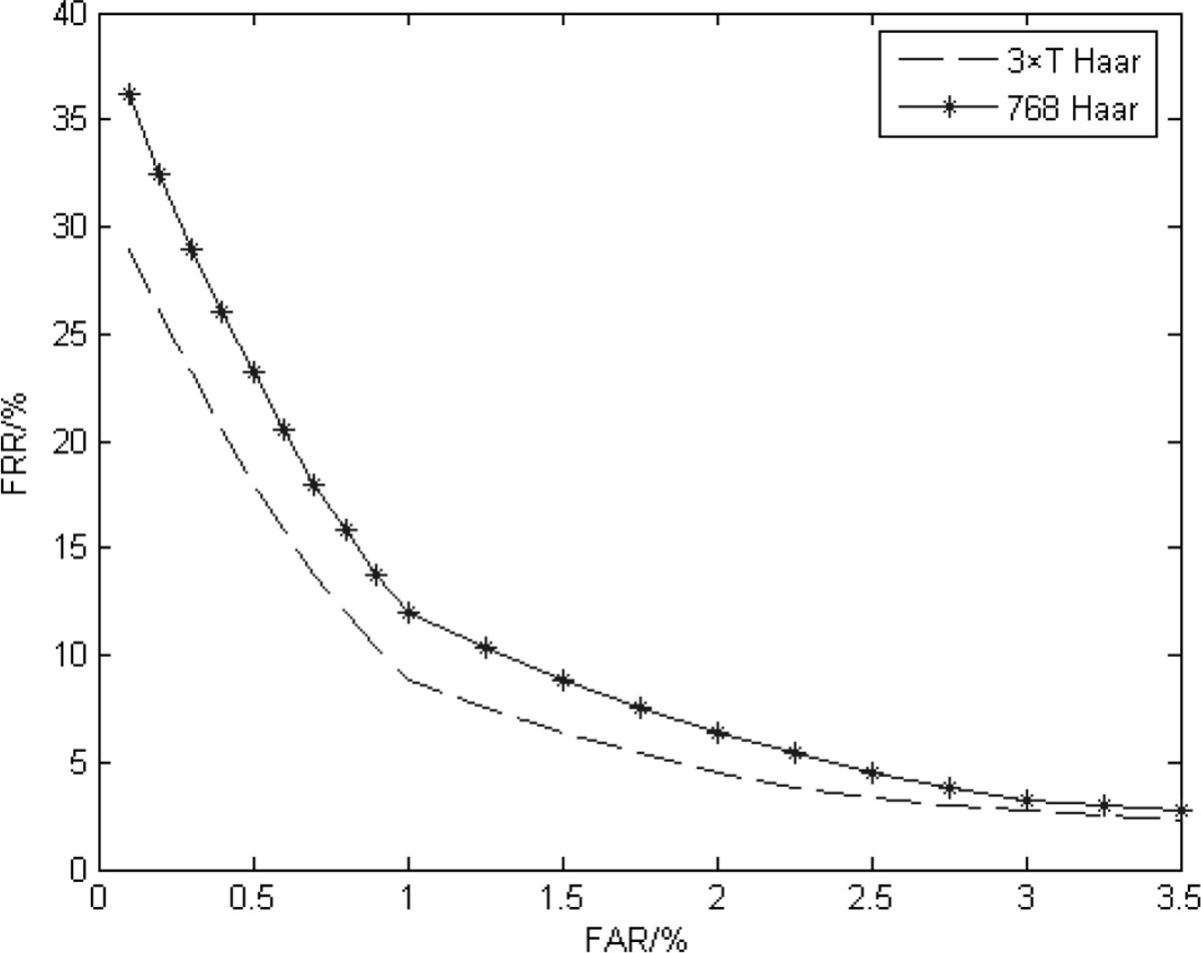
The ROC curve of the Haar wavelet experiment.

**Haar wavelet algorithm:** The third layer high-frequency coefficient feature is extracted by the Haar wavelet, eventually forming a binary feature code of 768 (32×8 ×3) bits. The Hamming distance is used for identification. The 3×T bit binary feature code for SCSF in this paper is compared, and the effect of the SCSF mechanism on iris certification is observed. The CRR and EER of different methods are shown in Table 3, and the ROC curve is shown in Fig 11.

**Table 3:** The CRR and EER of different methods in the Haar wavelet experiment.

Method	CRR	EER
3×T Haar	97.13%	2.84%
768 Haar	95.97%	3.12%

**Fig 12:**
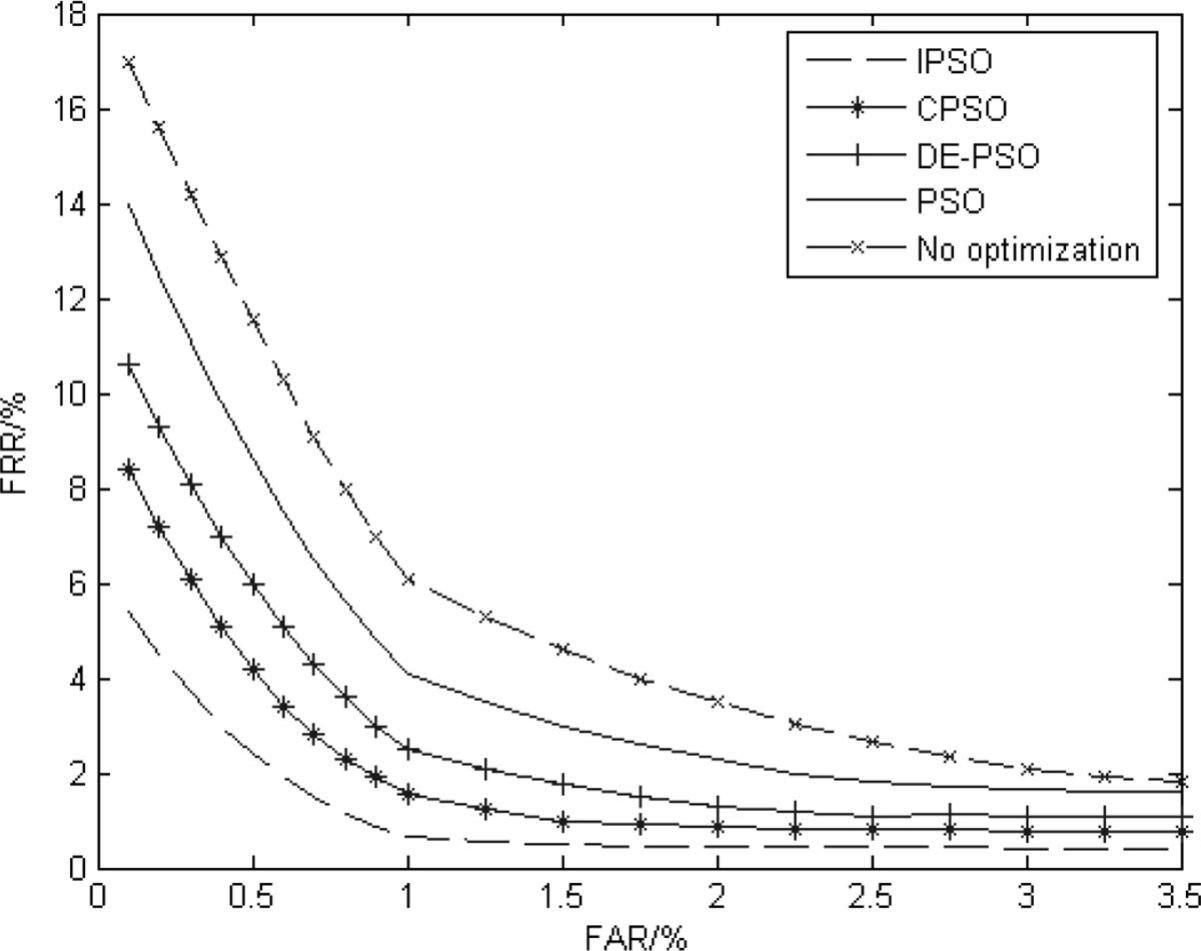
The ROC curve of the Gabor filter experiment.

**Gabor filter algorithm:** The Gabor filter with IPSO is compared with chaotic particle swarm optimization (CPSO) in the literature [4], differential evolution particle swarm optimization (DE-PSO) in the literature [39], general PSO algorithm, and the Gabor filter with no optimization. The Hamming distance is used for identification. The influences of different parameter optimization methods are observed. The initial values and optimization values of the Gabor filter parameters are shown in Table 4. The CRR and EER of different methods are shown in Table 5. The ROC curve is shown in Fig 12.

**Table 4:** The initial values and optimization values of Gabor filters.

Method	Initial value		Optimization value	
	*k*_max_	*f*^*v*^	*k*_max_	*f*^*v*^
IPSO	*1*.*352π*	*2*.*156*	*0*.*468π*	*1*.*532*
CPSO	*0*.*756π*	*2*.*365*	*0*.*968π*	*1*.*251*
DE-PSO	*1*.*158π*	*1*.*261*	*1*.*654π*	*1*.*843*
PSO	*1*.*689π*	*0*.*587*	*1*.*023π*	*1*.*452*
No optimization	*π*	1	——	——

**Table 5:** The CRR and EER of different methods in the Gabor filter experiment.

Method	CRR	EER
ES-LBP	98.71%	1.84%
SCCS-LBP	97.74%	2.38%
CS-LBP	97.37%	2.84%
LBP	95.71%	3.25%

**Comparison of similar algorithms:**The algorithm in this paper (multi-algorithm voting) is compared with the following algorithms:

1. Yao's improved 2D Log-Gabor filter algorithm [40];
2. Donald's improved algorithm for discrete cosine transform [41];
3. Zhu's iris pigmentation spot detection algorithm based on the bilinear template and block strategy [42].

**Fig 13:**
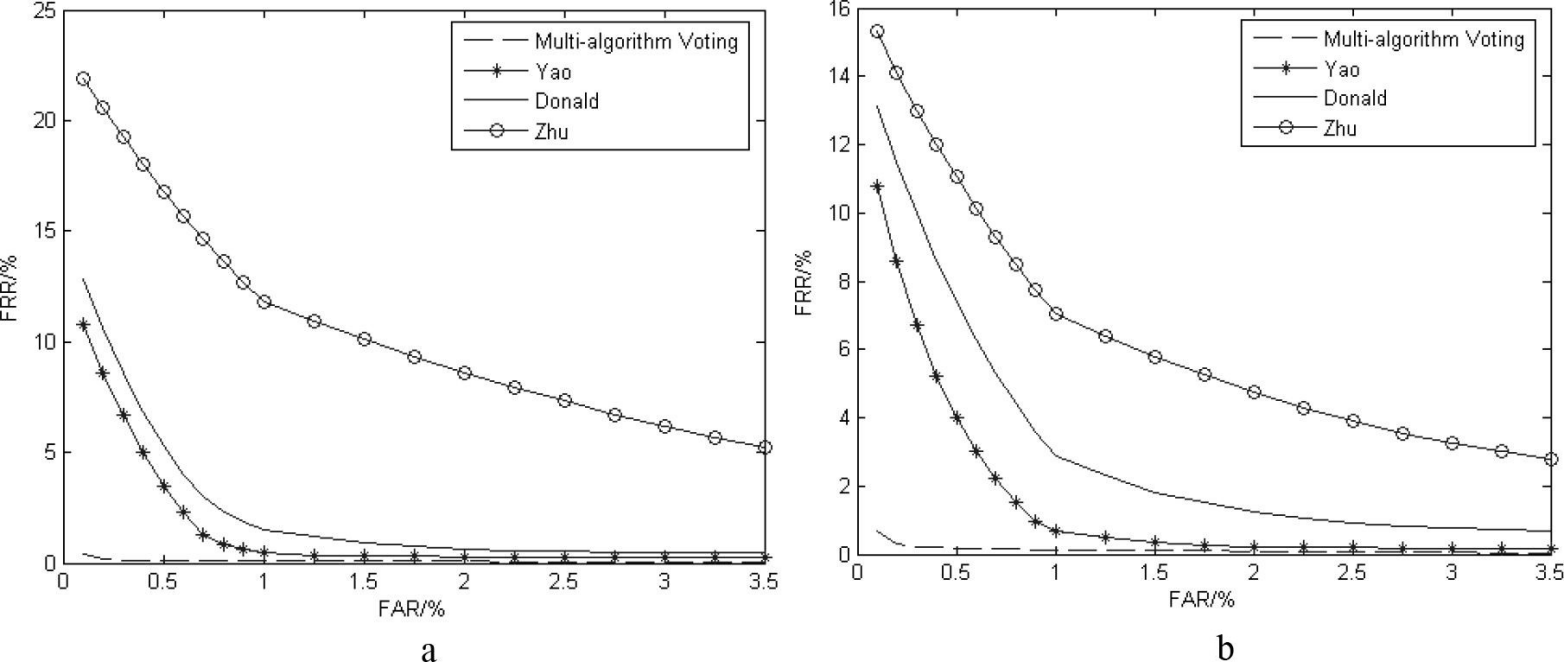
The ROC curve of traditional iris recognition algorithm experiment (a) CASIA-V4-Lamp, (b) CASIA-V4-Interval.

The number of iris images and the number of iris comparisons are shown in Table 6. The CRR and EER of the traditional iris certification algorithms experiment are shown in Table 7, and the ROC curve is shown in Fig 13.

**Table 6:** The number of iris images and the number of iris comparisons.

	Category number	Image number in each category	Total	Match number in the same category	Match number in different category	Total
Lamp	411	10	4110	3000	15000	18000
Interval	200	5	1000	1000	5000	6000

**Table 7:** The CRR and EER of the comparison of similar algorithms experiment.

Iris	CASIA-Iris-Lamp		CASIA-Iris-Interval	
Method	CRR	EER	CRR	EER
Multi-algorithm Voting	99.997%	0.18%	99.993%	0.23%
Yao	99.237%	0.805%	99.084%	0.925%
Donald	98.573%	1.215%	97.956%	1.634%
Zhu	91.865%	4.235%	94.568%	3.123%

The results of Fig 10 and Table 2 show that compared with the feature extraction of the general LBP algorithm, the other three algorithms can reduce the dimensions and storage space; however, the feature reduction ratios of SCCS-LBP and CS-LBP are too large, which omits some important texture information. The ES-LBP algorithm takes the stable feature points as the centre and extracts features in multiple surrounding circles. The feature extraction is more comprehensive, so the advantage is obvious on CRR.

It can be seen from the results of Fig 11 and Table 3 that compared with the method of Haar wavelet extraction for the entire enhancement region, the method of threshold detection for only feature points is better on CRR because the change in acquisition state leads to the change in iris texture state and then causes the iris feature relative relationship to weaken. The essence of iris feature extraction is to express the relative relationship in some form. Therefore, traditional algorithms for encoding the entire enhanced region are likely to increase the redundancy. On the basis of not considering the phase change, the SCSF only encodes the characteristic changes of stable feature points. Therefore, under the premise that the number of feature points is sufficient, the interference ability against the external state changes is better, which is more conducive to iris certification.

Table 5 and Fig 12 show that the certification effects using the optimization algorithm are better than the unoptimized certification effect. They indicate that the Gabor filter parameter setting has an influence on the certification, and it is necessary to optimize parameters. The particle swarm optimization algorithm can be used to optimize the parameters, but easily falls into a local optimal situation. CPSO, DE-PSO, and IPSO are designed to expand the search scope and better search globally. However, the search ranges of CPSO and DE-PSO are all in an uncertain state. Although there is a possibility of jumping out of local optimum, the original scope may not be completely searched and changing the search area to perform a new search will waste search space. The IPSO establishes a large search range, and the test iris is taken as an antigen. By observing the relationship between the test iris and the training iris, the full space can be searched completely.

It can be seen from Table 7 and Fig 13 that compared with the traditional excellent iris recognition algorithms, the SCSF multi-voting mechanism has the highest recognition accuracy and good robustness and stability, and the EER overall remains within the low level.

Because the algorithm in this paper is designed for the constrained iris with a long shooting interval, the most challenging problems affecting the recognition accuracy are limited to the defocus and external illumination during shooting. The iris phase deviation is negligible, so point-to-point precise certification can be achieved. Under the premise of SCSF, this paper's single algorithms all have advantages because the compression and amplification of the image causes the image's features to blur. Therefore, the process of searching for SCSF can exclude the interference of external redundancy and allow feature expression to be more focused on the relationship among stable features. The introduction of multiple feature expression algorithms improves the discrimination of different kinds of features. In a small number of categories, the distance class algorithm such as the Hamming distance can be used for certification to speed up certification. In summary, under the iris prerequisites of this paper, the existence of SCSF can effectively improve the accuracy of a single algorithm for small sample iris certification.

### 4.2. Multi-algorithm voting specific operation

According to the experiments of Section 4.1, the multi-algorithm voting method has practical significance. Therefore, for the specific voting situation, the irises are selected from JLU-4.0, CASIA-V1, CASIA-V4-Lamp, CASIA-V4-Interval, and CASIA-V4-Twin iris library that meet this paper’s prerequisites to constitute test iris library. When testing, use the same number of test iris in the same category for the experiment (the test iris is different from the training iris).

The number of images and matching situation are shown in Table 8. The number of correct certifications and the voting status under the multi-algorithm voting mechanism are shown in Table 9.

**Table 8:** The number of images and matching situation.

	Category number	Image number in each category	Total	Match number in different category	Match number in the same category	Total
JLU	250	200	50000	431562	102531	534093
V1	108	20	2160	63510	5325	68835
Lamp	411	40	16440	168628	28625	197253
Interval	200	15	3000	103640	10826	114466
Twin	100	80	8000	131532	18752	150284

**Table 9:** The number of correct authentication and the voting status.

	The same category					Different category				
	Correct	3 votes	2 votes	1 vote	0 vote	Correct	3 votes	2 votes	1 vote	0 vote
JLU	102530	100321	2209	1	0	431560	0	2	65	431495
V1	5323	5219	104	2	0	63509	0	1	41	63468
Lamp	28620	28495	125	4	1	168628	0	0	50	168578
Interval	10824	10738	86	2	0	103637	1	2	43	103594
Twin	18752	18667	85	0	0	131532	0	0	37	131495

Table 9 shows that compared with the single iris feature extraction algorithm, the voting mechanism of multiple algorithms can avoid recognition errors caused by defects in the image and algorithm. The defect of one algorithm can be compensated for by another algorithm. In the case of judging the same category, two vote situations indicate that one of three algorithms consider the test iris and the contrast iris to belong to different categories, such as the JLU Iris library, and the case of two votes is 2209. In the case of different categories of comparison, one vote situation exists, such as the JLU Iris library, and the case of one vote is 65. These results show that a multi-algorithm mechanism can effectively avoid judgement error, thereby increasing certification accuracy and expanding the range of recognizable irises. In addition, the Hamming distance algorithm also ensures certification speed.

### 4.3. Comparative experiments of traditional algorithms

Under the prerequisites of iris set in this paper, experiments are performed with a small sample iris set. By comparing with the iris certification algorithm trained on the ideal iris set, which shows that the algorithm in this paper causes unsteady iris caused by long time intervals on the accuracy of certification. Training iris and testing iris were selected from JLU-6.0.

The training iris of all algorithms is 1000 (20 categories of training iris, 50 training images of each category). During training, the training iris of the same category recognizes each other 2000 times, and the training iris of different categories recognizes each other 10,000 times. The number of test iris is also 1000 (20 categories of test iris, 50 test images in each category). The training iris is the same as the test iris category but the iris images are not the same and exclude extreme cases of interference. During test, the test iris of the same category recognized each other 1,000 times, and the test iris of different categories recognized each other 5000 times, a total of 6000 times.

Compare this method with the following seven algorithms:

1. Statistical learning model of convolutional neural networks based on VGG16 model architecture [43],explore the comparison of certification under the classic CNN architecture;
2. Multi-feature fusion certification algorithm based on Hamming distance in literature [6], compared with the distance class algorithm under multi-angle feature fusion;
3. Dynamic radial basis function neural network iris certification based on log-Gabor [44], compared with the traditional neural network algorithm statistical mode;
4. Ideal iris evidence theory certification by clustering method [45], compared with certification model under clustering feature of ideal iris set;
5. Concept cognition based on deep learning neural network [46], compared with deep learning architecture cognitive model under a small number of samples;
6. Iris recognition based on symmetric local binary pattern of statistical characteristic centers (SCCS-LBP) in literature [9], compared with spatial grayscale features in statistical mode;
7. Iris feature extraction and recognition based on image enhancement [47], compared with enhanced texture statistical recognition mode under unstable iris features;

The CRR and EER of the eight algorithms are shown in Table 10.

**Table 10:** CRR and EER of comparative experiments of traditional algorithms.

Method	The number of correct recognition	CRR
Algorithm in this paper	5993	99.883%
VGG16 model architecture	5547	92.450%
Multifeature fusion certification	4971	82.850%
Dynamic radial basis function neural network	5231	87.183%
Evidence theory certification	5621	93.683%
Deep learning neural network	5023	83.717%
SCCS-LBP	4426	73.767%
Image enhancement	5475	91.250%

The VGG16 model architecture is a commonly used convolutional neural network architecture and has been widely used in pattern recognition, but it is not suitable for the prerequisites of this paper. Because the texture state of iris images taken at long time interval is unpredictable, it is difficult to bring out the advantages of the VGG16 model.

The multifeature fusion certification algorithm combines two feature extraction algorithms, Haar wavelet and LBP algorithm, and authenticates irises by Hamming distance. In the ideal state iris sets, the differences among categories of iris are obvious, so the certification accuracy is very high. However, in the non-ideal state iris sets, the feature differences among many categories of iris are not obvious, and the Hamming distance is suitable when there is large feature difference and lower certification rate.

Dynamic radial basis neural network iris certification based on log-Gabor is a dynamic parameter test based on the results between the test and training irises in a certain period of time. Although the algorithm can be adjusted dynamically for different iris states through dynamic adjustment, due to the lack of a corresponding feedback mechanism, the algorithm adjustment is too dependent on labor, which results in a certification accuracy rate that is not as good as the algorithm in this paper.

The evidence theory cognitive model is used to find the common points of training iris data according to the clustering method, and then determine the iris features, which can better reflect the cluster points in steady state iris images. However, it is relatively difficult to select cluster points for unsteady state irises and this method also has certain requirements for the number of steady-state features, resulting in decreased certification accuracy.

The deep learning algorithm depends on the amount of data and the classification of data labels. When the data sample size is small and the data are not classified, the training effect of deep learning is poor, which will cause a poor recognition effect.

The iris feature extraction algorithm based on image enhancement can be used to highlight iris textures by image edge detection. The algorithm can extract features more carefully; however, the image quality needs to meet certain requirements. Feature extraction is difficult for unsteady state iris(such as defocused iris), which can make the certification accuracy lower than that of the algorithm in this paper.

From all, it can be concluded that the algorithm in this paper has certain advantages in the expression and recognition of unsteady state iris texture caused by long time interval shooting under small samples.

### 4.4. Time operation experiment

Under the prerequisites of this article, the images were selected from JLU-4.0, JLU-6.0, CASIA-V4-Lamp, CASIA-V4-Interval iris library to constitute the experimental iris library. The iris in the experimental iris library has passed the test of the quality evaluation algorithm to ensure that the pre-processing stage will not affect the iris recognition. The classification thresholds of the three algorithms in the recognition model are trained by 1000 training iris images (50 categories are selected from each iris library and 20 images are from each category). The test iris used a series of 10 additional images in the same category (500 in each iris library) for continuous authentication experiments. Take experiments to test all times (in milliseconds (ms)) and accuracy, which are shown in Table 11.

**Table 11:** The result of time operation experiment.

Iris Library	Algorithm running time in this paper	The number of correct recognition
JLU-4.0	2356ms	498
JLU-6.0	2167ms	500
CASIA-V4-Lamp	2542ms	497
CASIA-V4-Interval	2459ms	199

It can be seen from Table 11 that after 500 consecutive certification experiments of different images, the running time of different iris banks is within 2100-2600ms. In the case of ensuring that the front-end preprocessing is completed, the certification time of a single image is 4-5ms, which meets the actual work requirements.

## 5. Conclusion

In this paper, certain algorithm improvement attempts are made for the case of unstable features caused by unsteady-state iris images. For the same category of fast certification problem in the case where accurate statistical learning cannot be performed on the data set and data status, a one-to-one fast certification algorithm for constrained unsteady-state iris based on the scale change stable feature and multi-algorithm voting is proposed The equal-dimensional differential Gaussian space is constructed to search for stable region feature points. Then, three algorithms are proposed to perform iris certification from three aspects of the filter method, spatial domain method and image transformation method. It is determined whether the two irises belong to the same category according to the voting result. While ensuring the accuracy of algorithm certification, the algorithm can improve the anti-interference of the texture information unsteady caused by the acquisition state change. The experiments show that the proposed algorithm has practical application significance and improves certification accuracy, robustness and certification speed.

The prerequisites for the algorithm involved in this paper are still relatively harsh, and the range of available recognition is also small. How to further improve the recognition accuracy and realize multi-categories recognition on the basis of expanding algorithm prerequisites will be the next research focus.
